# Renal function and lipid metabolism in Japanese HIV-1-positive individuals 288 weeks after switching from tenofovir disoproxil fumarate to tenofovir alafenamide fumarate: a single-center, retrospective cohort study

**DOI:** 10.1186/s40780-024-00336-y

**Published:** 2024-02-28

**Authors:** Kensuke Abe, Junji Imamura, Akiko Sasaki, Tomoko Suzuki, Satomi Kamio, Taku Obara, Toshihiro Ito

**Affiliations:** 1 Department of Pharmacy, National Hospital Organization Morioka Medical Center, 1-25-1 Aoyama, Morioka, Iwate, 020-0133 Japan; 2 Department of Clinical Research, National Hospital Organization Sendai Medical Center, 983-8520, Miyagino 2-11-12, Sendai, Japan; 3 HIV/AIDS Comprehensive Medical Center, National Hospital Organization Sendai Medical Center, 983-8520, Miyagino 2-11-12, Sendai, Japan; 4 Department of Pharmacy, National Hospital Organization Shibukawa Medical Center, 377-0280, Shirai 383, Shibukawa, Japan; 5 Department of Pharmaceutical Scienses, Tohoku University Hospital, 980-8574, Seiryomachi 1-1, Sendai, Japan

**Keywords:** Renal function, Lipid metabolism, HIV, Tenofovir disoproxil fumarate, Tenofovir alafenamide fumarate

## Abstract

**Background:**

Continued use of tenofovir disoproxil fumarate (TDF), an antiretroviral drug, causes renal function decline and tubular damage in individuals with HIV. While tenofovir alafenamide fumarate (TAF) may have less damaging effects, it causes weight gain and abnormal lipid metabolism.

**Methods:**

This single-center, retrospective cohort study used medical records from the National Hospital Organization Sendai Medical Center to investigate renal function of Japanese HIV-1-positive individuals who switched from TDF to antiretroviral therapy including TAF by 2017. The endpoints were: estimated glomerular filtration rate (eGFR), urinary β2 microglobulin (Uβ2MG), weight, and lipid metabolism parameters at 288 weeks after switching. Possible correlation between eGFR and Uβ2MG and factors affecting eGFR decline were examined.

**Results:**

Sixty patients switched from TDF to TAF and continued therapy for 288 weeks. eGFR showed a significant decline after 144 weeks, although it was controlled from the time of change until 96 weeks. In the renal impairment group, the decline was suppressed until week 288. Uβ2MG continued to decrease significantly after 48 weeks. However, the suggested correlation between eGFR and Uβ2MG disappeared when patients switched from TDF to TAF. Weight and lipid metabolic parameters increased significantly at 48 weeks and were maintained. Factors associated with decreased eGFR were: history of acquired immune deficiency syndrome (AIDS) and Uβ2MG. However, considering the odds ratio, the switch from TDF to TAF suppressed the eGFR decline in the group with a history of AIDS, and Uβ2MG had no effect on the eGFR decline.

**Conclusions:**

Switching from TDF to TAF for the long term slows eGFR decline, decreases Uβ2MG levels, and reduces worsening of renal function. Weight gain and abnormal lipid metabolism may occur in the short term but are controllable.

## Introduction

Tenofovir disoproxil fumarate (TDF), an antiretroviral drug, is a prodrug of tenofovir (TFV), a nucleoside reverse transcriptase inhibitor (NRTI) that exerts antiviral effects by inhibiting human immunodeficiency virus (HIV) reverse transcriptase [1]. However, the continued use of TDF is known to decrease renal function and cause tubular damage [2]. The drug is taken up from blood into tubules, passed through the proximal tubular cells of the kidney, and excreted into urine [3]. During this process, TFV is concentrated in the tubular cells, resulting in damage [4].

In contrast, tenofovir alafenamide fumarate (TAF), another prodrug of TFV, is less likely to be taken up by renal tubular cells because of its high stability in plasma and cell membrane permeability, which activates TFV in cells, such as cluster of differentiation 4(CD4) + T cells, targeted by HIV [5]. As a result, TAF has a more potent antiviral effect than TDF at doses less than one-tenth that of TDF [6]. Therefore, it is expected to have less of an effect on renal tubular cells than TDF and to reduce renal tubular damage [7]. Japanese HIV-1-positive individuals taking antiretroviral therapy including TDF for at least 48 weeks and continuously for 144 weeks after switching to TAF showed reduced renal function decline [8].

In the Japanese anti-HIV treatment guidelines [9], TAF has been recommended as a first-line drug for NRTIs, along with TDF, since 2016 [10]. TDF is no longer a first-line drug and has been recommended based on clinical circumstances since 2018 [11]. Comparative studies based in North America [12, 13], Africa [14], Taiwan [15], and Europe [16] as well as a pooled analyses of these studies [17] reported that HIV-positive individuals showed increased weight or body mass index (BMI) and abnormalities in lipid metabolism after the use of TAF. Japanese HIV-1-positive individuals also had significantly worse low-density lipoprotein cholesterol (LDL-cho) and triglyceride (TG) levels, as well as a significant increase in weight 12 months after switching to TAF [18]. In the Japanese population, the risk factors of eGFR decline including increased age and elevated TG, LDL-cho, and BMI levels [19] are significantly associated with obesity and chronic kidney disease (CKD) [20].

Therefore, in order to confirm the long-term safety of TFV-containing drugs, especially TAF, we evaluated the renal function of Japanese HIV-1 positive patients after switching from antiretroviral therapy including TDF to TAF. Furthermore, we assessed the changes in their body weight and lipid metabolism following long-term TAF administration.

## Methods

### Study design and patients

This single-center, retrospective cohort study used medical records from the National Hospital Organization Sendai Medical Center in Sendai, Tohoku, Japan. The study population consisted of Japanese HIV-1-positive individuals aged ≥ 18 y who had switched from antiretroviral therapy, including TDF (300 mg/day) to TAF (25 mg/day or TAF 10 mg/day; the latter for antiretroviral therapy regimens including cobicistat or ritonavir) by December 2017.

We examined the effect of antiretroviral therapy including TDF on their renal function for at least 48 weeks. The rationale behind setting the 48-week threshold was based on a randomized, open-label, non-inferiority trial (Study 934) that reported the efficacy as well as adverse events of the treatment at 48 weeks [21]. In addition, individuals who took TAF continuously for 288 weeks were included in the study to evaluate renal function, weight, and lipid metabolism after switching from TDF to TAF for a long period of time.

The eligibility criteria for conversion from TDF to antiretroviral therapy including TAF in this study were: 1) serum creatinine (SCr) consistently > 1.2 mg/dL (the upper limit of the reference value at our hospital); and 2) an abnormally high urinary β2-microglobulin (Uβ2MG) (≥ 10,000 μg/L) after TDF administration. In addition, individuals who had no obvious renal dysfunction but were explained and agreed to switch from TDF to TAF by their physician/pharmacist to prevent worsening of renal function from continued TDF use were also included. For the third class of medications, a non-NRTI (NNRTI), protease inhibitor (PI), or integrase inhibitor (INSTI) was used in combination with TDF or TAF. The exclusion criteria for participants after switching from TDF to TAF were as follows: 1) death within 288 weeks of starting TAF; 2) transfer to other hospitals; 3) discontinuation of TAF medication; and 4) significant adherence problems due to continued irregular visits to our hospital.

This research was approved by the Clinical Research Department and Ethics Committee of the National Hospital Organization Sendai Medical Center in May 2019 with registration numbers No.31–93 and C31-86. The requirement for patient consent was waived by the ethics committee due to the retrospective nature of the study. The study conformed to the Declaration of Helsinki of the World Medical Association, the Ethical Guidelines for Life Sciences and Medical Research Involving Human Subjects, and the Act on the Protection of Personal Information.

### Measurements

The index of renal function related to the primary endpoint was estimated glomerular filtration rate (eGFR) by SCr, as recommended by the Japanese Society of Nephrology. The formula is eGFR (mL/min/1.73 m^2^) = 194 × [SCr]-1.094 × [age]-0.287 × [0.739 for women] [22]. The eGFR classification for different stages of CKD was based on the "KDIGO 2012 clinical practice guideline for the evaluation and management of chronic kidney disease" [23]. The indices of weight and lipid metabolism related to the secondary endpoints were BMI (kg/m^2^), TG (mg/dL), total cholesterol (T-cho) (mg/dL), high-density lipoprotein cholesterol (HDL-cho) (mg/dL), and LDL-cho (mg/dL). BMI was calculated using the formula, BMI (kg/m^2^) = [body weight] × [height]^−2^ [24].

To characterize the population, HIV-1 ribonucleic acid (RNA) viral load (copies/mL) and amount of CD4 (cells/μL) were identified as indicators of HIV suppression and immune status. Other investigative variables included age, sex, history of acquired immunodeficiency syndrome (AIDS), third-class medications for antiretroviral therapy, duration of TDF administration, urinary protein, and history of hypertension, diabetes mellitus, or dyslipidemia.

Laboratory values for the primary and secondary endpoints were based on the time of switch from TDF to TAF (week 0, TAF0). Values at 48 (TAF48), 96 (TAF96), 144 (TAF144), 192 (TAF192), 240 (TAF240), and 288 (TAF288) weeks after the switch were employed for statistical analysis. The primary endpoint was the comparison of eGFR and Uβ2MG values at each point from TAF48 to TAF288, using TAF0 values as reference, to evaluate the impact on renal function after switching from TDF to TAF. The secondary endpoints were comparisons between BMI values and TG, T-cho, HDL-cho, and LDL-cho values at each time point to evaluate the impact of switching from TDF to TAF on weight and lipid metabolism. Blood samples were drawn in the morning, and the test results included HIV-1-positive individuals who needed to take anti-retroviral medications after breakfast and HIV-1-positive individuals who had consumed a meal.

### Statistical analysis

eGFR (mean ± standard deviation (SD)) was compared using a paired t-test at each point with TAF0 as the reference. The duration of TDF administration (mean ± SD) in each group after classification into GFR categories was compared using Student's t-test. Uβ2MG (median; IQR) was compared using Wilcoxon's signed rank test at each point with TAF0 as the reference. The correlation between eGFR and Uβ2MG was confirmed via Spearman's rank correlation coefficient and single regression analysis at TAF0 and TAF288, respectively. BMI (mean ± SD), TG (mean ± SD), T-cho (mean ± SD), HDL-cho (mean ± SD), and LDL-cho (mean ± SD) were compared using paired t-test at each point with TAF0 as reference. Finally, since the average annual eGFR decline rate in the Japanese population is estimated to be 0.36 mL/min/1.73 m^2^ [25], multiple logistic regression analysis was performed to predict factors that influenced a greater decline in eGFR, setting the criteria for the eGFR decline rate at TAF288 at an average of 2.00 mL/min/1.73 m^2^ [≒ 0.36 mL/min/1.73 m2 × 5.5 years (288 weeks)]. All statistical analyses were performed using JMP®, version 14.2 (SAS Institute, Cary, North Carolina, USA) with a significance level of 0.05.

## Results

### Study population

As of December 2017, there were 170 HIV-1-positive individuals who were regular attendees at the National Hospital Organization Sendai Medical Center, of whom 125 were being treated with antiretroviral therapy including TAF. Of the 125 individuals, 18 who were treated with TAF from the start and 11 whose previous therapy was antiretroviral therapy including abacavir were excluded from the study. In addition, one non-Japanese person and five patients who had switched to TAF after receiving TDF for less than 48 weeks were excluded. Finally, 90 individuals were included in the study. By 288 weeks, which was set as the study period, 27 individuals had been transferred to other hospitals due to work commitments, one individual died after being diagnosed with HIV encephalopathy, one had his hospital visit interrupted due to disappearance, and one switched to a two-drug therapy involving Dolutegravir (DTG)/Lamivudine (3TC) combination from cobicistat (COBI) boosted Darunavir (bDRV)/Emtricitabine (FTC)/TAF combination about 211 weeks after starting TAF to reduce the size and number of components of the pills. Therefore, 60 individuals who continued antiretroviral therapy including TAF for the entire 288 weeks were included in the analysis. As shown in Table 1, HIV-1-positive individuals were taking a variety of the third class of medications when they started taking TDF, but when they switched from TDF to TAF, 51.7% of the 60 population individuals were taking DTG. As others, 23.3% were taking COBI boosted Elvitegravir (EVG), 16.7% were taking Raltegravir (RAL), and 8.3% were taking bDRV. None of the participants switched the third class of medications to that of another for 96 weeks after switching from TDF to TAF. However, with the launch of the Bictegravir (BIC)/FTC/TAF combination in April 2019, the number of HIV-1-positive individuals who switched from each of the third class of medications to BIC increased after 144 weeks. Finally, at 288 weeks, 66.7% of the 60 population individuals was taking BIC. Otherwise, 11.7% were taking DTG, 3.3% were taking EVG, 8.3% were taking RAL, 6.7% were taking bDRV, and 3.3% were taking Doravirine (DOR).

**Table 1:**
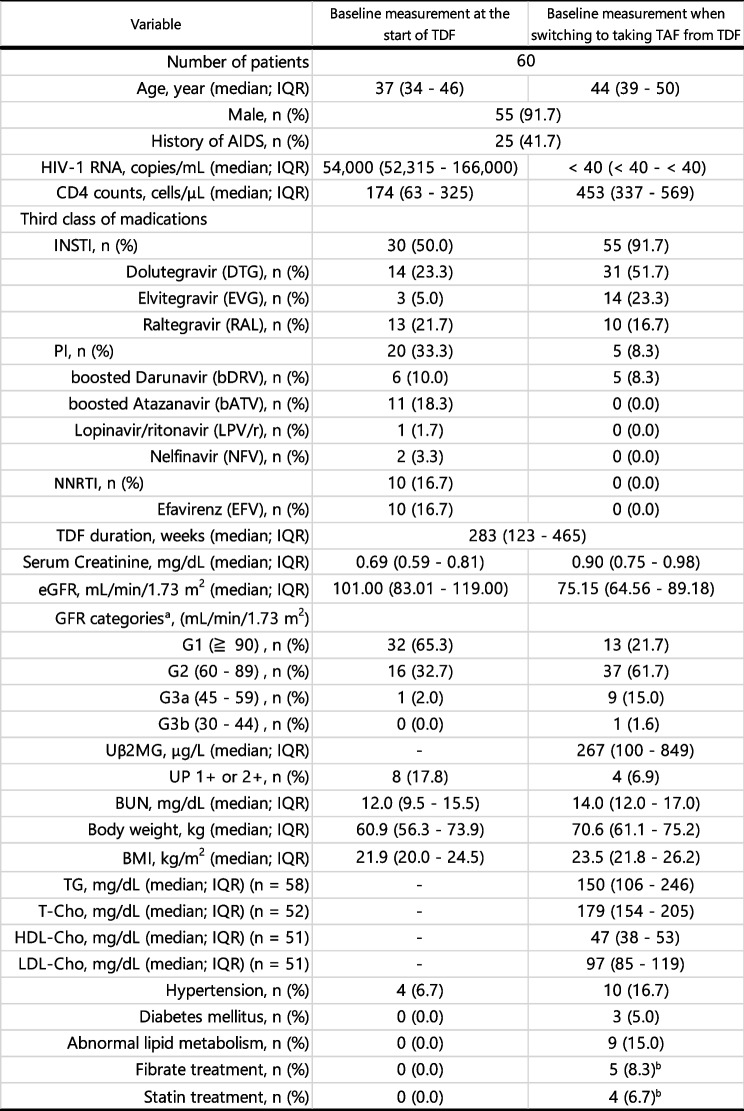
Characteristics of Japanese HIV-1-positive individuals in the study

*IQR* Interquartile range

^a^No subjects classified as G4 and G5

^b^No patient took these drugs together

Table 1 shows the characteristics of the 60 individuals. At the switch to antiretroviral therapy including TAF, most of the analyzed patients had good viral control and improved immunity with a median CD4 count of 453 (IQR: 337–569) cells/μL. Percentage values of the third class of medications used in combination with TDF or TAF are also included in Table 1. The median duration of treatment with TDF was 283 weeks (IQR: 123–465 weeks), which is comparable to the 288 weeks of treatment with TAF in this study. The median SCr and eGFR were 0.90 (IQR: 0.75–0.98) mg/dL and 75.15 (IQR: 64.56–89.18) mL/min/1.73 m^2^, respectively, at the switch from TDF to TAF. Additionally, the median Uβ2MG, which was not measured at the start of TDF therapy, was 267 (IQR: 114–869) μg/L. Similarly, the median body weight and median BMI were 70.6 (IQR: 61.1–75.2) kg and 23.5 (IQR: 21.8 – 26.2) kg/m^2^, while the median TG, T-cho, HDL-cho, and LDL-cho levels were 150 (IQR: 106–246), 179 (IQR: 154–205), 47 (IQR: 38–53), and 97 (IQR: 85–119) mg/dL, respectively. Subjects who were receiving medications for hypertension, diabetes, and lipid metabolic disorders at the start of TDF and TAF are shown in Table 1.

### Change in renal function

**Fig. 1:**
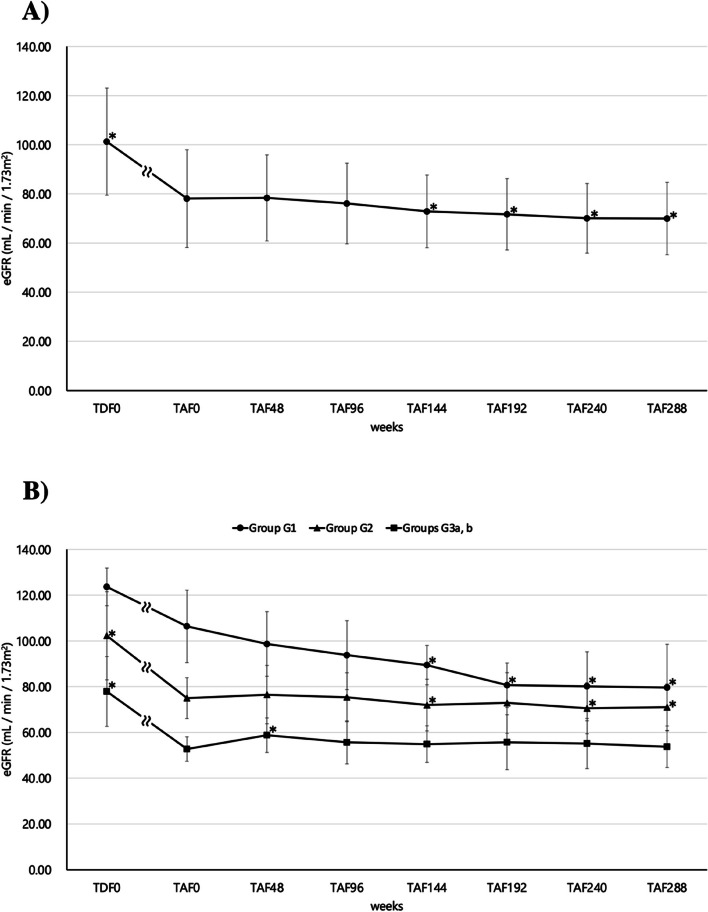
**A**) Change in eGFR (mean ± SD) over 288 weeks in HIV-positive individuals who switched from TDF to antiretroviral therapy including TAF and continued taking it. **B** Change in eGFR (mean ± SD) over 288 weeks in each group classified by eGFR level at the time of switching from TDF to TAF (week 0, TAF0). In both (**A**) and (**B**), paired t-tested was performed at 48 weeks (TAF48), 96 weeks (TAF96), 144 weeks (TAF144), 192 weeks (TAF192), 240 weeks (TAF240), and 288 weeks (TAF288), using TAF0 as the reference, with a significance level of *p* < 0.05. The sample size of B) at each survey from TAF0 to TAF288 was 13 in group G1, 37 in group G2, and 10 in groups G3a, b. At TDF0, there were 8 in group G1, 32 in group G2, and 9 in groups G3a, b

The eGFR changes in the 60 patients are shown in Fig. 1A. From TDF0 to TAF0, eGFR showed a significant decrease (mean difference (MD) = 23.20 mL/min/1.73 m^2^, 95% confidence interval (CI) = 19.05–29.26, *p* < 0.0001). No significant differences remained in TAF48 and TAF96 levels after switching to antiretroviral including TAF. The eGFR after TAF144 (MD = -5.20 mL/min/1.73m^2^, 95% CI: -8.69 – -1.72, *p* = 0.0041) was significantly lower than that of TAF0.

Figure 1B shows the changes in eGFR classified into three groups based on GFR categories according to the eGFR values at TAF0. There were no G4 or G5 patients in this study. TDF durations (mean ± SD) for groups G1 (291 ± 158 weeks), G2 (296 ± 212 weeks), and G3a, b (311 ± 188 weeks) showed no significant difference.

After switching from TDF to TAF, the eGFR in group G1 continuously decreased for up to 288 weeks, with a particularly significant decrease after week 144 (MD = -16.96 mL/min/1.73 m^2^, 95% CI: -29.65 – -4.27, *p* = 0.0130). In group G2, the decline in eGFR was suppressed until 96 weeks, finally showing a significant decrease (MD = -4.11 mL/min/1.73 m^2^, 95% CI: -7.69 – -0.53, *p* = 0.0256) at 288 weeks. In contrast, in groups G3a and b, eGFR significantly increased at 48 weeks (MD = 6.06 mL/min/1.73 m^2^, 95% CI: 1.87–10.26, *p* = 0.0097) compared to that at TAF0. After 96 weeks, the inhibition of eGFR decline continued until week 288, although this difference was not statistically significant.

**Fig. 2:**
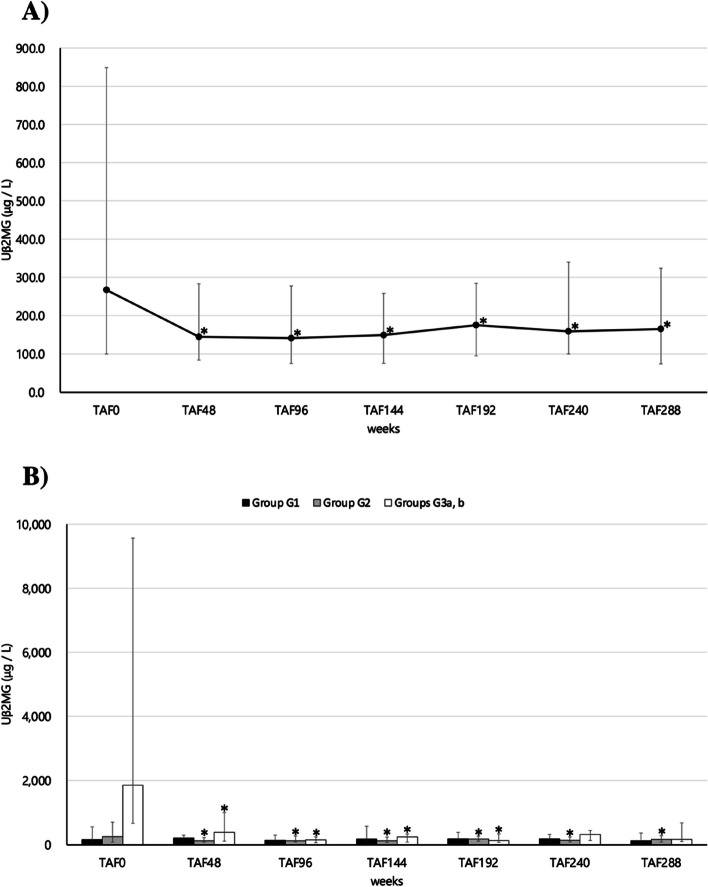
**A**) Change in Uβ2MG (median; IQR) over 288 weeks in HIV-positive individuals who switched from TDF to antiretroviral therapy including TAF and continued taking it. **B**) Change in Uβ2MG (median; IQR) over 288 weeks in each group classified by eGFR level at the time of switching from TDF to TAF (week 0, TAF0). In both (**A**) and (**B**), Wilcoxon signed rank test was performed at 48 weeks (TAF48), 96 weeks (TAF96), 144 weeks (TAF144), 192 weeks (TAF192), 240 weeks (TAF240), and 288 weeks (TAF288), using TAF0 as the reference, with a significance level of *p* < 0.05. The sample size of B) at each survey from TAF0 to TAF288 was 11 in group G1, 33 in group G2, and 7 in groups G3a, b

**Fig. 3:**
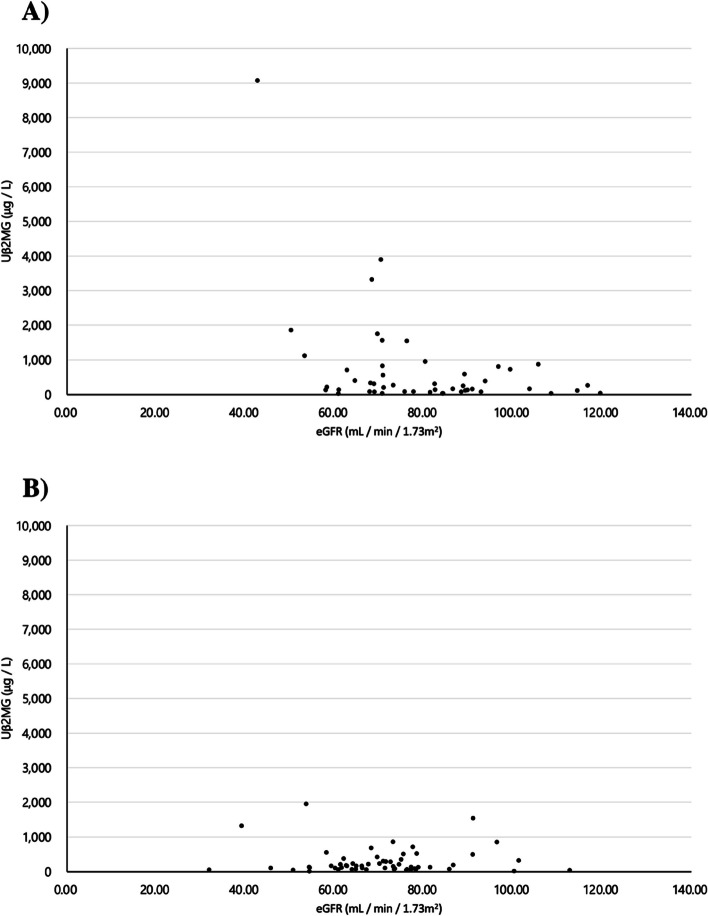
Scatter plots of eGFR and Uβ2MG at (**A**) week 0 (TAF0) and (**B**) week 288 (TAF288) for 60 subjects

Changes in Uβ2MG are shown in Fig. 2A as an indicator of renal tubular damage. Uβ2MG significantly decreased at 48 weeks (MD = -2753.5 μg/L, 95% CI: -6471.9 –—964.8, *p* < 0.0001) compared to TAF0 and remained significantly lower thereafter until TAF288 (MD = -2700.2 μg/L, 95% CI: -6273.9 –—873.4, *p* = 0.0013). The changes in Uβ2MG in groups G1, G2, and G3a, b based on GFR classification are shown in Fig. 2B. After switching from TDF to TAF, Uβ2MG in the G3a and b groups decreased significantly at TAF48 (MD = -14,881.0 μg/L, 95% CI: -44,606.0 –—14,843.5, *p* = 0.0156), and the significant decrease was maintained thereafter until TAF192 (MD = -15,335.0 μg/L, 95% CI: -44,914.0 –—14,243.5, *p* = 0.0156). However, there was no significant difference at TAF240 and TAF288. In the group G2, Uβ2MG significantly decreased at 48 weeks (MD = -866.8 μg/L, 95% CI: -2024.4 –—290.8, *p* = 0.0125) compared to TAF0, and the decrease was maintained thereafter until TAF288 (MD = -917.4 μg/L, 95% CI: -2050.9 –—216.2, *p* = 0.0146). In the group G1, Uβ2MG was originally low at TAF0 after the switch from TDF to TAF, and there was consistently no significant difference up to TAF288. On testing the association between eGFR and Uβ2MG at TAF0 and TAF288, Spearman’s rank correlation coefficient was -0.3859 (*p* = 0.0052) at TAF0 and 0.0587 (*p* = 0.6643) at TAF288. The results of the single regression analysis were *p* = 0.0320 at TAF0 and *p* = 0.8914 at TAF288. Thus, a negative correlation between eGFR and Uβ2MG was suggested when taking antiretroviral therapy, including TDF; however, this association disappeared on switching to TAF (Fig. 3A and B).

### Changes in BMI and lipid metabolism parameters

**Fig. 4:**
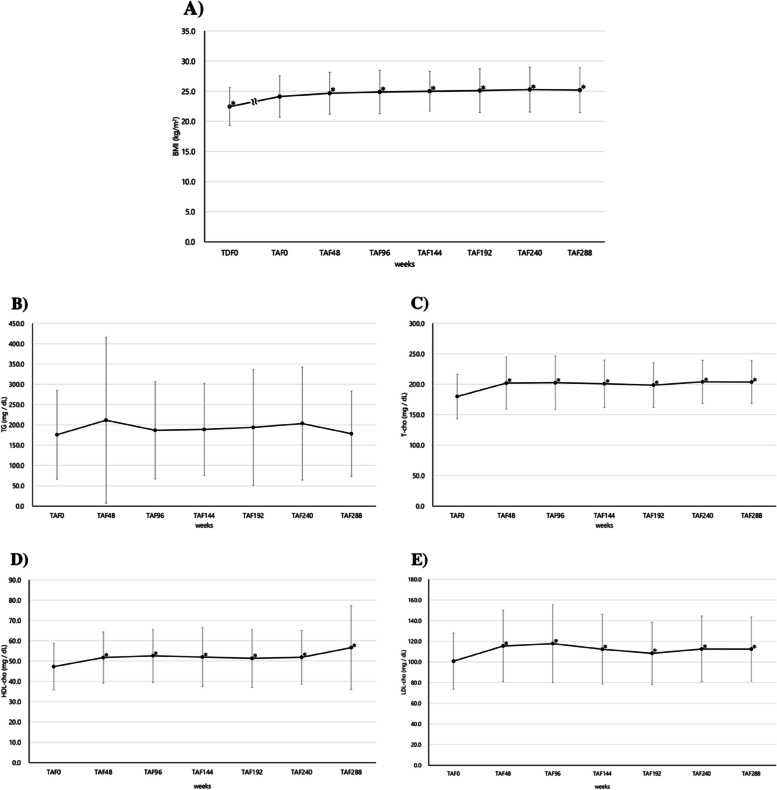
Changes in (**A**) BMI (mean ± SD), (**B**) TG (mean ± SD), (**C**) T-cho (mean ± SD), (**D**) HDL-cho (mean ± SD), and (**E**) LDL-cho (mean ± SD) over 288 weeks of individuals who switched from TDF to antiretroviral therapy including TAF and continued taking it. In **A**), **B**), **C**), **D**), and **E**), paired t-test was performed at 48 weeks (TAF48), 96 weeks (TAF96), 144 weeks (TAF144), 192 weeks (TAF192), 240 weeks (TAF240) and 288 weeks (TAF288), with TAF0 as the reference, and the significance level was set at *p* < 0.05

Figure 4A shows BMI as an indicator of weight. BMI showed a significant increase at each time point after 48 weeks (MD = 0.56 kg/m^2^, 95% CI: 0.26–0.87, *p* = 0.0005) compared to that at TAF0. Thereafter, it remained near the upper reference limit of 25.0 kg/m^2^ [24] until TAF288 (MD = 2.50 kg/m^2^, 95% CI: 1.63–3.37, *p* < 0.0001). Additionally, a comparison between TAF48 and TAF288 showed no significant difference.

Figures 4B, C, D and E show changes in TG, T-cho, HDL-cho, and LDL-cho as parameters of lipid metabolism. The TG levels were already above the reference level (50–149 mg/dL) at the time of switching from TDF to TAF. The TG levels at each point were compared with those at TAF0, all of which continued to show no significant differences. T-cho showed a significant increase at 48 weeks (MD = 22.1 mg/dL, 95% CI: 13.6–30.7, *p* < 0.0001) when compared to TAF0 at each point and then remained within the reference levels (150–219 mg/dL) until TAF288 (MD = 23.8 mg/dL, 95% CI: 13.2–34.4, *p* < 0.0001). HDL-cho showed a significant increase at 48 weeks also (MD = 4.5 mg/dL, 95% CI: 2.4–6.5, *p* < 0.0001) when compared to TAF0 at each time point and then remained within reference levels (men: 40–86 mg/dL, women: 40–96 mg/dL) until TAF288 (MD = 9.4 mg/dL, 95% CI: 4.0–14.7, *p* = 0.0009). Moreover, LDL-cho showed a significant increase at 48 weeks (MD = 14.7 mg/dL, 95% CI: 8.5–20.8, *p* < 0.0001) when compared to TAF0 at each point and remained within reference levels (70—139 mg/dL) until TAF288 (MD = 11.7 mg/dL, 95% CI: 4.3–19.2, *p* = 0.0027). A comparison between TAF48 and TAF288 showed no significant differences in any of the aforementioned parameters.

### Factors associated with changes in eGFR

The prediction results of factors affecting the change in eGFR at 288 weeks after switching from TDF to TAF are shown in Table 2. The outcome was whether the decline in eGFR between TAF0 and TAF288 was ≥ 2.0 mL/min/1.73 m^2^, and the associated factors that might reduce eGFR above the mean annual rate of decline in the Japanese population were identified. Factors affecting the decrease in eGFR were: history of AIDS (odds ratio (OR) = 0.1361, 95% CI: 0.0205–0.9015, *p* = 0.0232) and Uβ2MG (OR = 0.9996, 95% CI: 0.9992–0.9999, *p* = 0.0087). However, when these ORs are considered, switching from TDF to TAF affected the suppression of eGFR decline in the group with a history of AIDS. Switching from TDF to TAF also reduced Uβ2MG and had little effect on eGFR decline.

**Table 2:**
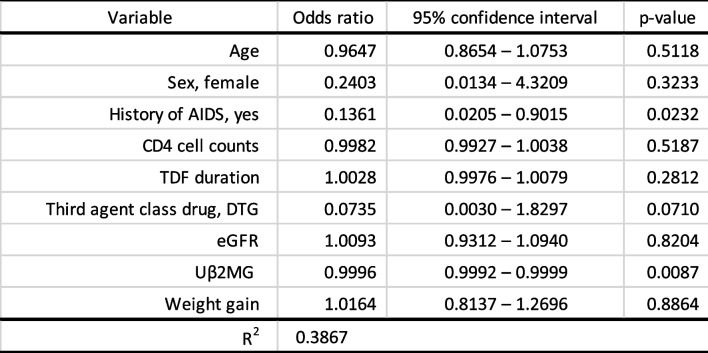
Results of multiple logistic regression analysis predicting factors affecting mean annual decline in eGFR greater than or equal to 2.00 mL/min/1.73 m^2^

## Discussion

At 288 weeks after switching from TDF to TAF, there was a significant decrease in eGFR in the population; however, in the group with lower renal function, the decrease in eGFR was controlled early and maintained until 288 weeks. Uβ2MG also declined early and was maintained until 288 weeks. Renal dysfunction induced by TDF in Japanese HIV-1-positive individuals was associated with renal tubular damage, whereas the damage was improved and less likely to occur with TAF. Thus, our results suggest that antiretroviral therapy including TAF less likely to cause renal dysfunction. With regard to body weight and lipid metabolism, there was a significant increase in BMI, T-cho, HDL-cho, and LDL-cho at 288 weeks after switching from TDF to TAF. However, these were significantly increased at 48 weeks, followed by no significant difference between 48 and 288 weeks. Finally, factors affecting the decline in eGFR included a history of AIDS and Uβ2MG, which were implicated in preventing the decline in eGFR.

The effects of TAF on renal function have been reported worldwide. First, a pooled analysis of 26 previously published reports showed that creatinine clearance decreased less significantly from baseline to 96 weeks in the TAF group compared to the TDF group, and for Uβ2MG, it increased from baseline in the TDF group but decreased in the TAF group [26]. The Swiss HIV Cohort Study examined changes in eGFR and urine protein/creatinine ratio after 18 months in HIV-positive individuals who switched from TDF to TAF, and in those who did not. The results of this study showed that switching from TDF to TAF improved eGFR and proteinuria in patients with renal dysfunction [27]. In Japan, TAF has been reported to reduce renal function decline and tubular damage at 144 weeks and 12 months in HIV-positive individuals who switched from TDF to TAF [8, 18]. However, to the best of our knowledge, the present study is the first to report an observation of renal function over a long period of 288 weeks after switching from TDF to TAF. Based on our findings, we consider antiretroviral therapy including TAF a long-term treatment option even for HIV-positive individuals with impaired renal function.

Weight gain and abnormal lipid metabolism have been reported with the use of TAF. First, a cohort study on weight change in HIV-positive individuals who switched from TDF to TAF in the United States showed that all HIV-positive individuals had a significant annual weight gain of approximately 1.80–4.47 kg soon after the switch. However, they also reported that weight gain tended to slow down or stagnate approximately 9 months after the switch [13]. The Swiss Cohort Study also compared weight and lipid metabolism and reported adverse changes such as weight gain, development of obesity, and worsening serum lipid levels in those who continued with TAF [16]. In a recent report from Japan, after a switch from TDF to TAF, and after more than 2 years of follow-up on weight and lipid metabolism, the annual weight change was comparable between the TDF and TAF treatment periods. However, this report showed that weight gain was observed in HIV-positive individuals who received both TAF and DTG and in those younger than 50 years [28]. The results of our study on BMI and lipid metabolism parameters after switching from TDF to TAF are possibly the first data in the world to present solid longitudinal changes over a period of 288 weeks. Our findings are similar to those of previous studies as significant increases in BMI and lipid metabolic parameters were observed in the short term within 48 weeks after the switch, followed by a slow long-term course. In our department, the first priority for HIV-positive individuals with weight gain and elevated lipid metabolism parameters is dietary guidance by a nutritionist rather than the immediate administration of hyperlipidemia medications such as statins and fibrates. None of the patients received additional hyperlipidemia medications within 288 weeks of initiating antiretroviral therapy including TAF. We consider this therapy as a controllable long-term treatment with respect to weight gain and lipid metabolism.

Our study had some limitations. It was a single-center study with a small sample size of only Japanese patients, some of whom had their third class of medicines switched during the course of treatment. Moreover, the administration of antiretroviral drugs that inhibit organic cation transporter 2 and multidrug and toxin extrusion 1, such as DTG [29], BIC [30], and COBI in combination with EVG [31] and DRV [32], may have affected the decrease in eGFR associated with an increase in SCr. However, as shown in Table 1, the percentage of HIV-1-positive individuals taking their antiretroviral drugs at the time of the switch from TDF to TAF was 83.3% in total, but did not change significantly to 88.4% at 288 weeks after the switch to TAF. In addition, the HIV-1-positive individuals included subjects whose blood was drawn after breakfast, so the lipid-related data may have been affected somewhat by meals. Our study may also have been limited by the exclusion of the 65 individuals for various reasons described in the section titled Study population under Results. However, we believe that the exclusion reference levels we established were reasonable in terms of bias, although all laboratory values for the primary and secondary endpoints were accumulated without missing values for the 288 weeks we set, and the number of cases was reduced by appropriate analysis.

## Conclusions

In Japanese HIV-1-positive individuals, long-term antiretroviral therapy including TAF can be safely used without damage to renal function. In particular, it was suggested that switching to TAF may reduce the eGFR decline in patients with a history of AIDS. The switch from TDF to TAF may result in weight gain and elevated lipid metabolic parameters, but these issues can be managed in the long term. Future studies may incorporate data from multiple centers with a larger sample size to validate our current findings, including those from countries other than Japan.

## Data Availability

All data generated or analyzed in this study are included in this paper.

## Abbreviations

TDF: Tenofovir disoproxil fumarate
TFV: Tenofovir
NRTI: Nucleoside reverse transcriptase inhibitor
HIV: Human immunodeficiency virus
TAF: Tenofovir alafenamide fumarate
CD4: Cluster of differentiation 4
BMI: Body mass index
LDL-cho: Low-density lipoprotein cholesterol
TG: Triglyceride
CKD: Chronic kidney disease
SCr: Serum creatinine
Uβ2MG: Urinary β2-microglobulin
NNRTI: Non-nucleoside reverse transcriptase inhibitor
PI: Protease inhibitor
INSTI: Integrase inhibitor
eGFR: Estimated glomerular filtration rate
T-cho: Total cholesterol
HDL-cho: High-density lipoprotein cholesterol
HIV-1 RNA: Human immunodeficiency virus-1 ribonucleic acid
AIDS: Acquired immunodeficiency syndrome
SD: Standard deviation
IQR: Interquartile range
DTG: Dolutegravir
3TC: Lamivudine
COBI: Cobicistat
bDRV: Boosted Darunavir
FTC: Emtricitabine
EVG: Elvitegravir
RAL: Raltegravir
BIC: Bictegravir
DOR: Doravirine
MD: Mean difference
CI: Confidence interval
OR: Odds ratio
